# Operating Room Performance Optimization Metrics: a Systematic Review

**DOI:** 10.1007/s10916-023-01912-9

**Published:** 2023-02-04

**Authors:** Anne M. Schouten, Steven M. Flipse, Kim E. van Nieuwenhuizen, Frank Willem Jansen, Anne C. van der Eijk, John J. van den Dobbelsteen

**Affiliations:** 1 Biomedical Engineering Department, Technical University of Delft, Mekelweg 5, 2628 CD Delft, the Netherlands; 2 Science Education and Communication Department, Technical University of Delft, Mekelweg 5, 2628 CD Delft, the Netherlands; 3 Gynecology Department, Leiden University Medical Center, Albinusdreef 2, 2333 ZA Leiden, the Netherlands; 4 Operation Room Centre, Leiden University Medical Center, Albinusdreef 2, 2333 ZA Leiden, the Netherlands

**Keywords:** Operation Room, Optimization, Workflow, Performance Metrics

## Abstract

Literature proposes numerous initiatives for optimization of the Operating Room (OR). Despite multiple suggested strategies for the optimization of workflow on the OR, its patients and (medical) staff, no uniform description of ‘optimization’ has been adopted. This makes it difficult to evaluate the proposed optimization strategies. In particular, the metrics used to quantify OR performance are diverse so that assessing the impact of suggested approaches is complex or even impossible. To secure a higher implementation success rate of optimisation strategies in practice we believe OR optimisation and its quantification should be further investigated. We aim to provide an inventory of the metrics and methods used to optimise the OR by the means of a structured literature study. We observe that several aspects of OR performance are unaddressed in literature, and no studies account for possible interactions between metrics of quality and efficiency. We conclude that a systems approach is needed to align metrics across different elements of OR performance, and that the wellbeing of healthcare professionals is underrepresented in current optimisation approaches.

**Supplementary Information:**

The online version contains supplementary material available at 10.1007/s10916-023-01912-9.

## Background

Operating Room (OR) performance optimization is investigated from many angles and numerous different strategies are proposed. Think hereby of new systems based on data analysis that enable more efficient OR scheduling. However, many of these promising initiatives that are meant to improve the OR do not seem to land in practice [1]. Suggested changes do not always fit the overall workflow of the OR, or they solve the targeted problem ineffectively. Creating a support base amongst the people that implement or work with the innovation also tends to be problematic [2]. To enable improvement of OR performance with innovations that fit well in practice, it should be clear what is exactly meant with the term *OR performance.* Furthermore, to know if an innovation improves overall OR performance, one must know how to measure the overall performance. Below, we discuss what OR performance means according to literature and which elements it contains. Next, to investigate how to measure OR performance we make an inventory of the metrics used in literature to measure OR performance. Finally, to investigate how the field approaches OR performance optimization, we collected studies on this topic and addressed what methods were used and what aspect of OR performance the research focussed on. Besides perspectives on patients and healthcare professionals, we also consider economic perspectives on the OR on hospital budgets.

### Pressures for change in the OR

The OR comprises a complex environment with multi-layered social interactions, unpredictability and a low tolerance for mistakes [3]. Irregularities in the workflow are often triggered by a combination of factors such as demanding caseloads, pressure to perform complex tasks and conflicting priorities. This can result in increased mental strain and stress amongst the healthcare professionals [4].

Irregularities in workflow on the OR also impact patients. Approximately 60% of the patients visit the OR at some point during their hospital stay [5]. Undergoing hospital admission and an operation makes many people experience emotions such as nervousness, agitation and uncertainty. Irregularities in the process can worsen this [6].

Accounting for about 35% to 40% of the costs, the OR is a large contributor to a hospital’s finances, also being one of the most costly units [7–9]. Over the past years, healthcare costs have increased and diminishing returns have prompted healthcare administrators to alleviate institutional costs through reductions in budget allocations [10].

Partly driven by the increasing demands for care on the one hand, and constrained resources on the other, a technological evolution has taken place over the last decades. This has played an important role in the development of surgery and resulted in dramatic changes in working conditions within the OR [11]. But healthcare professionals are not always prepared for this transformation of their work. Healthcare professionals are reported to lack preparation for radical (technical) changes in their work [12].

Despite the growing influx of new healthcare professionals, the sector experiences a major exodus of healthcare professionals. Causes are the heavy workload and a lack of autonomy. The limited autonomy of healthcare professionals in their daily work is appointed as a long-standing issue [12]. Research from the Dutch doctors organisation *De Jonge Dokter* has interviewed 622 young doctors about their work. About 50% of the interviewees has thought about quitting their job due to high work pressure, emotional pressure and working culture [13].

The impact of the workflow of the OR on patients, pressure on healthcare professionals that work on the OR, the vast changing work environment and economical constraints make that optimizing the OR is high on the academic agenda. However, the high expectations of patients, interactions between different professionals, unpredictability and complex surgical case scheduling make managing and changing the system difficult. Attempts to resort to commonly used industrial principles to increase factors such as efficiency have been demonstrated to easily fail due to these (and possibly other) particular characteristics of the OR [7]: human factors have too great of an impact to standardize and automate certain OR processes. Another complicating factor is the divergent perspectives on OR performance optimization.

### OR performance optimization metrics

The metrics used to quantify OR performance optimization reported in literature are diverse [14]. Many articles focus on the efficiency aspect of OR performance optimization, some focus more on the quality aspects. For example, the work of Bellini et al. [7] speaks of the optimization related factor efficiency in the sense of more precise scheduling and limiting waste of resources. Costa Jr. et al. (2015) speak of both efficiency and optimization, hereby focussing on resources and time management. Sandbaek et al. [16] refer to OR efficiency as maximizing throughput and OR utilization while minimizing overtime and waiting time, without additional resources. Tanaka et al. (2011) assesses OR performance using indicators such as the number of operations, the procedural fees per OR, the total utilization time per OR and total fees per OR. Rothstein & Raval [3] refer to the metrics of OR efficiency based on the Canadian Paediatric Wait Times Project: off-hours surgery, same-day cancellation rate, first case start-time accuracy, OR use, percentage of unplanned closures, case duration accuracy, turnover time and excess staffing costs. Alternatively, Arakelian, Gunningberg and Larsson (2008) emphasize that apart from cost-effectiveness, work in the OR should be organized to fulfil the demands on patient safety and high-quality care. From their perspective, OR departments must create efficient ways of planning and processing the work, while at the same time maintaining the quality of care. These authors also show that there are diverging perspectives among OR personnel on what efficiency and productivity entail.

The previous paragraphs illustrate that when speaking about OR performance optimization, different terminology is being used. Furthermore, although many studies focus on how to optimize or monitor certain aspects of the OR, studies on the impact of these changes on the quality and efficiency of the hospital as a whole appear to be lacking. This may lead to uncertain optimisation strategies that are difficult to substantiate with supporting evidence [15]. Table 1 summarizes both quality and efficiency aspects of OR workflow and strives to align the methods and metrics to assess OR performance in terms of 1. Patient safety 2. Quality of care 3. Cost-effectiveness and 4. Healthcare professional well-being.

**Table 1:** OR performance includes four aspects: 1. Patient safety 2. Quality of care 3. Cost-effectiveness 4. Well-being healthcare professionals

OR performance	
Efficiency *Maximizing throughput and OR utilization while minimizing overtime and waiting time, without additional resources* [16]	Cost-effectiveness [17]
Quality	Quality of care [17]
	Patient Safety [17]
	Well-being healthcare professional [12]

## Method

A systematic literature review was conducted to make an inventory of metrics for optimization of the OR in literature. We used the search engines Scopus, Web of Science and PubMed with the search terms: “Operation Room” AND Optimization and Workflow AND Optimization AND Hospital. We limited the search to articles that discuss ways to optimize the OR as a system, not the performed medical interventions themselves. Furthermore, articles that were not written in the English language or did not belong to the category *healthcare* or *medicine* were excluded.

### Analysis

An inventory of the topics of the articles was made by filtering out 1. the focus/aim of the study, 2. the method used and 3. the conclusion. Optimization strategies in other hospital departments might be transferable to the OR as well. Therefore, to gain insight in the distribution of optimization strategies on the different departments of the hospital, the articles were analysed by labelling the operational *department* the research is focussed on, which *topic* was investigated, and which *method* was used. After creating this overview, only data about the OR was used. To obtain OR performance metrics a second analysis was conducted: OR performance characteristics from the articles were split into aspects with their corresponding metrics.

#### Coding nodes

Overall categories for departments, topics and methods were identified based on the first 50 articles, as the authors felt a saturation rate for new categories was reached. The remaining articles were then labelled within these categories. To illustrate, Table 2 shows two sections that were both labelled as the metric T_3 and two sections that were labelled as the method M_8.

**Table 2:** Sections from articles that were labelled as T_3 and sections that were labelled as M_8, to illustrate when these sections fall in the same category

Topic label T_3: Optimize patient flow	
*1*	“*In most hospitals, patients move through their operative day in a linear fashion, starting at registration and finishing in the recovery room. Given this pattern, only 1 patient may occupy the efforts of the operating room team at a time. By processing patients in a parallel fashion, operating room efficiency and patient throughput are increased while costs remain stable”* [18]
*2*	*“The main objective of this work is to propose and to evaluate a Decision Support System (DSS) for helping medical staff in the automatic scheduling of elective patients, improving the efficiency of medical teams’ work”* [5]
Method label M_8: Computational	
*1*	“*To solve the allocation of doctors to surgeries planning problem, also addressed in literature as Master Surgical Schedule (MSS), we propose a mathematical programming approach”* [19]
*2*	*“In this study, three optimization models were developed for optimizing operating room scheduling during unexpected events and accommodating emergency patient surgeries in the established schedule. The first model (Model I) schedules emergency patients in newly opened rooms, whereas the second model (Model II) aims to assign emergency patients to untapped ranges; the third model (Model III) re-sequences elective and emergency patients in the room with the greatest free margin”* [20]

#### Coding the articles

Some articles mention multiple topics or methods. If multiple topics were mentioned, the article was labelled for the topic which had the most emphasis. This is illustrated in Example 1, where both the topics patient throughput and costs are mentioned. However, the emphasis is on patient throughput. For topics the article was therefore labelled as T_3: Optimize patient flow.

When labelling the articles for their method, it occurred that an article investigates an optimization possibility and method by means of a literature study. The method of the article was then labelled as literature study. In Example 2, the article investigates how workflow can be improved by identifying the potential failures of the system by means of a management tool. However, the effects of the management system on workflow are investigated by a literature study. For methods the article was therefore labelled as M_1: Literature study. In the results the coded articles are displayed in 3 sunburst graphs: the first shows the distribution of methods and topics of all hospital departments, the second contains the distribution of methods and topics on the OR. To elucidate the OR data, the third graph shows a selection with the biggest categories of the second sunburst (categories with N ≥ 3). Some of the smaller categories are illustrated with examples in the text.

#### Validation

The labelling was performed independently by two of the authors. Discrepancies were discussed and adjusted by obtaining consensus.

Example 1: *“In most hospitals, patients move through their operative day in a linear fashion, starting at registration and finishing in the recovery room. Given this pattern, only 1 patient may occupy the efforts of the operating room team at a time. By processing patients in a parallel fashion, operating room efficiency and patient throughput are increased while costs remain stable” *[18].

Example 2: *“Failure mode and effects analysis (FMEA) is a valuable reliability management tool that can pre-emptively identify the potential failures of a system and assess their causes and effects, thereby preventing them from occurring. The use of FMEA in the healthcare setting has become increasingly popular over the last decade, being applied to a multitude of different areas. The objective of this study is to review comprehensively the literature regarding the application of FMEA for healthcare risk analysis” *[21].

## Results

In this section, the results of the inventory of OR performance metrics and the addressed OR performance topics in literature are shown.

### Review statistics

**Fig. 1:**
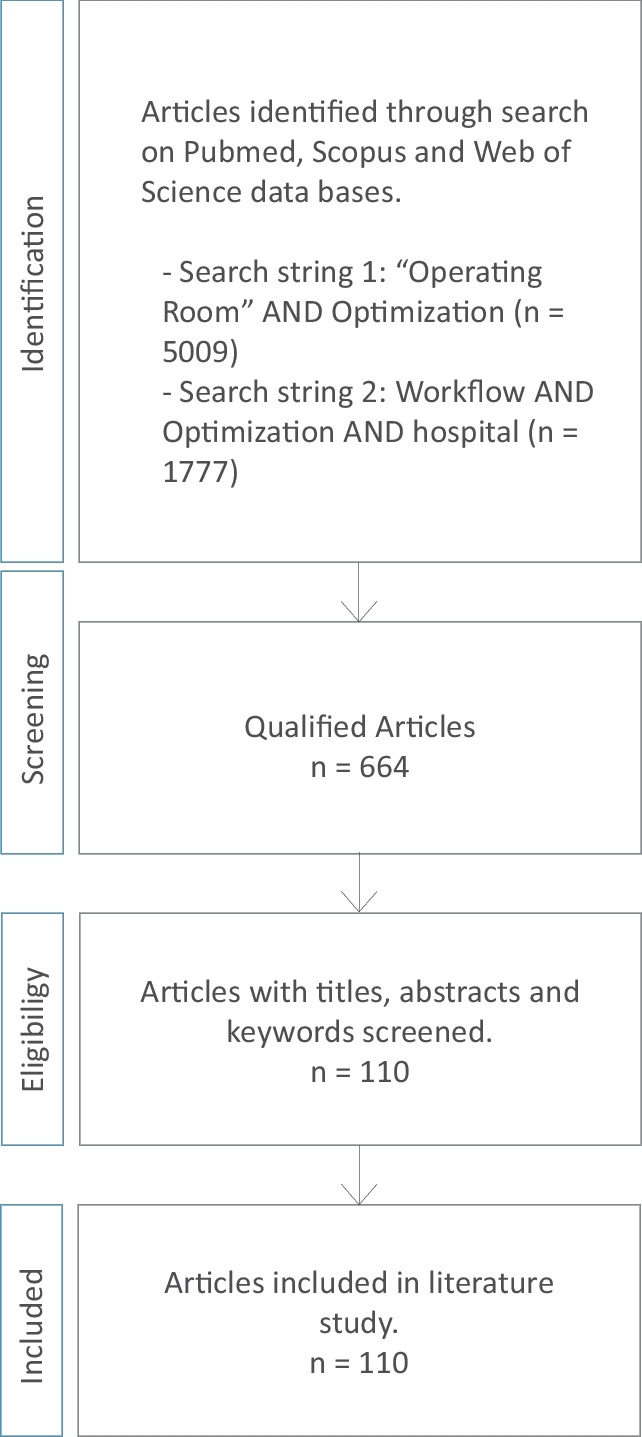
Literature search method used to make an inventory of the current literature on OR optimization

Figure 1 shows the search engines, search terms and number of papers found.

### OR performance metrics

Based on Table 1, the characteristics of OR performance (efficiency and quality) have been split up into aspects and were then further specified into metrics (Table 3).

**Table 3:** Characteristics, aspects, and metrics of OR performance as reported in literature

*Characteristic*	*Aspect*	*Source*	*Metric*	*Source*
Efficiency	OR throughput	[16]	Number of operations per OR per month	[22]
	OR utilization	[16]	Total utilization time per OR per month (hours)	[22]
	Time (overtime, waiting time)	[16]	Off-hours surgery per OR per month (hours) *(Results from urgent add-on cases or case over-runs) *	[3]
			Same-day cancellation rate per OR per month *(classify per cause and time of day.) *	[3]
			First case start-time accuracy per OR per month (%)	[3]
			OR use per OR per month (hours) *(Distinguish between overall utilization time (time something is occurring on the OR) and operating-specific utilization (time between first incision and final closure as a percentage of the room’s overall “open” period.) *	[3]
			Percentage of unplanned closures per OR per month (%) *(may occur due to equipment deficits *etc*.) *	[3]
			Case duration accuracy per OR per month (%) *(useful to distinguish between true case time (from patient entry till patient exit) and turnover times) *	[3]
			Turnover time per OR per month (hours) *(from patient exit till next patient entry) *	[3]
			Excess staffing costs per OR per month (€) *(can be the result of both over-utilization (pay staff for overtime) and underutilization) *	[3]
	Resources	[16]	Procedural fees per OR per month (€)	[22]
			Total fees per OR per month (€)	[22]
Quality	Quality of care & patient safety	[17]	Number of problems per patient as a result of exposure to the healthcare system per OR per month	[23]
	Well-being health care professionals	[12]	Workload *(number of cases per OR staff member per month) *	[12]
			Autonomy	[12]

### Addressed OR performance topics in literature

Table 4 shows the categories used to analyse the articles, the corresponding labels and their names. In Appendix 1, Supp. Tab. 1, the *topic* category and their corresponding sources are presented.

**Table 4:** The categories used to analyse the articles, corresponding labels and names

*Category*	*Label*	*Name*	*Description*
Department	D_1	OR	OR department
	D_2	ER	ER department
	D_3	Outpatient clinic	Outpatient clinic department
	D_4	Patient clinic	Patient clinic department
	D_5	Hospital	Hospital in general
Topic	T_1	Optimize role of surgeon	
	T_2	Reduce delays	
	T_3	Optimize patient flow	
	T_4	Reduce costs	
	T_5	Optimize management	Organisational management, risk management etc
	T_6	Optimize teamwork	
	T_7	Reduce non operative time	
	T_8	Optimize anaesthesia procedure	Can be optimization of both medication and procedure
	T_9	Define OR efficiency	
	T_10	Optimize scheduling	Scheduling of operations, medical staff etc
	T_11	Optimize overall equipment effectiveness	Use equipment in a more effective way
	T_12	Optimize workflow tracking systems	Such as an engineering perspective or VSM (Value Stream Mapping)
	T_13	Optimize overall productive capacity of a department	
	T_14	Optimize department design	Physical rearrangement or redesign of a department
	T_15	Reduce workload	
Method	M_1	Literature review	
	M_2	Analysing data	
	M_3	Experiment with patients	
	M_4	Experiment with surgeons	
	M_5	LEAN	Management strategy to strip a company or organization from unnecessary steps in their processes
	M_6	Experiment with department team	Specific team of a certain department
	M_7	Experiment with hospital staff	Includes medical staff (i.e., doctor, nurse) and other staff (i.e., managers, administration)
	M_8	Computational	Such as simulations with artificial intelligence
	M_9	OSH integrated risk management (Occupational Safety and Health)	
	M_10	System engineering	

### Distribution of the labels

Appendix 2, Supp. Figure 1, shows a sunburst graph that illustrates the distribution of the labels per department (D_x). To give an overview that represents the distribution of departments in a hospital, only the data from the second search criteria (Workflow AND Optimization AND Hospital) was included in this graph. The inner circle contains the different departments, namely the OR, ER (emergency room), outpatient clinic, patient clinic and the hospital in general. The middle circle shows the corresponding methods, the outer circle shows the topics. Most articles focus on the OR (D_1, N = 16). The ER receives less attention (D_2, N = 2). There were no articles that were labelled for outpatient clinic (D_3).

**Fig. 2:**
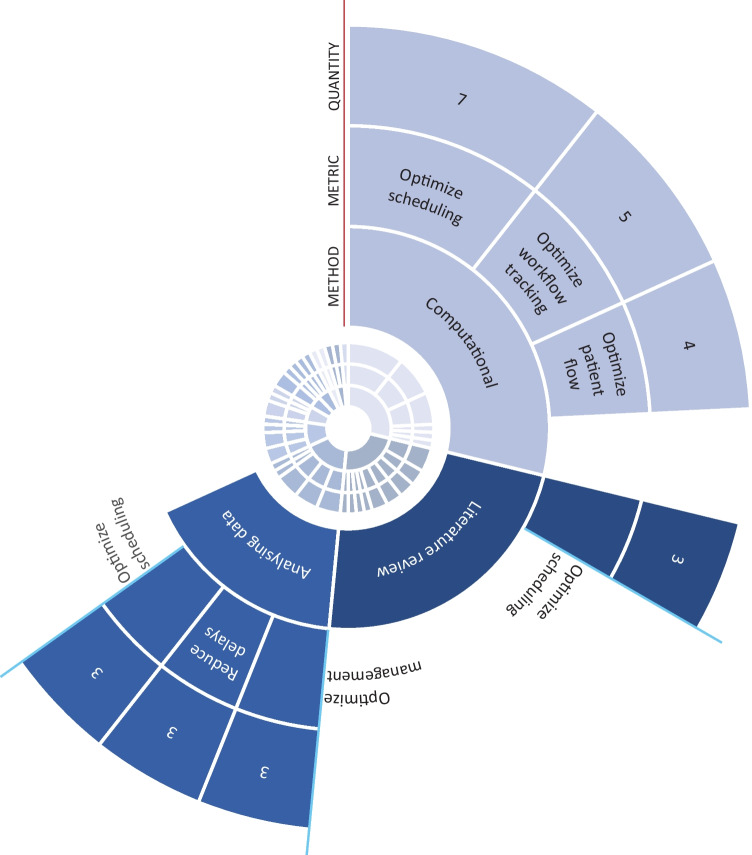
Selection of the sunburst graph, showing the seven main categories

In Appendix 2, Supp. Figure 2 zooms in on methods and corresponding topics of just the OR. This graph includes all OR data from both search criteria and shows the methods, topics and number of articles in each category. In Fig. 2, a selection of the OR sunburst graph is displayed. This selection contains the most frequent combination of period, method and topic (N ≥ 3). Most articles have the aim to optimize scheduling (N = 7), workflow tracking (N = 5) and patient flow (N = 4) by computational means such as machine learning.

**Fig. 3:**
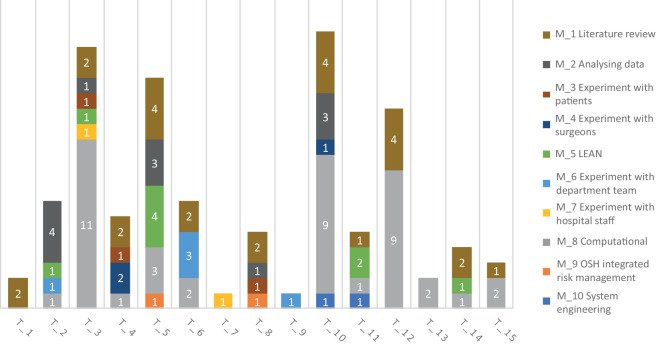
Bar chart with the different methods per topic

All data was then stratified by the means of a bar chart. Figure 3 shows the data while looking at different methods per topic. Computational methods (M_8) are used most frequently (N = 41).

The methods that were used the least are experiments with the medical staff (M_7, N = 2) and system engineering (M_10, N = 2). The most investigated topics are patient flow (T_10, N = 18), OR scheduling (T_3, N = 17) and workflow tracking systems (T_5, N = 15).

## Discussion

In this study we addressed what methods were used in other studies, what aspect of OR performance they focussed on, and for which department the effects were to be relevant. We aimed to investigate how the field approaches OR performance optimization and to create an overview of OR performance metrics for the categories of patient safety, quality of care, cost-effectiveness, and the well-being of healthcare professionals.

Most studies focused on patient safety, quality of care and cost-effectiveness. This might be explained by the fact that healthcare has a central focus on patient wellbeing and clinical outcome measures. One striking result from this study is that the well-being of healthcare professionals is largely ignored in OR optimization studies. Ill-performing in these areas may contribute to staff shortages. Therefore, we deliberately added the well-being of healthcare professionals as a crucial aspect of OR performance as we feel that this is a subject that must be taken in account in OR optimisation.

By taking all four categories within OR performance as a starting point for the delineation of the ways to measure OR performance, we strive to create an all-encompassing overview of relevant metrics in literature. More metrics were found for efficiency than for quality aspects. This was as expected because efficiency tends to be easier to measure than quality aspects. Furthermore, quality metrics are often subjective. For instance, *well-being of healthcare professionals,* is linked to the metric *autonomy* (freedom to make your own choices, plan your workday etc.)*.* This is a capacity that is difficult to quantify in a valid and reliable way.

Considering the research topics addressed in literature it was found that most articles have the aim to optimize *OR scheduling, Workflow tracking* or *Patient flow* by computational means, such as machine learning. Thanks to greater computing power, as well as the growing availability of large amounts of data, machine learning holds the promise to make sense of complex modelling tasks [24]. Topics such as *OR scheduling, Workflow tracking* and *Patient flow* fit this picture. They are suitable for computational simulations and optimizations of complex systems such as the OR that are characterised by high variability in the timing and alignment of processes.

Categories that involve experiments with healthcare professionals (such as interventions in practice) were only limitedly represented in the literature. With AI on the rise, it seems a logical choice to use simulations to test optimizations instead of occupying the (often overworked) healthcare professionals. However, although simulated efficacy trials have generated many possible interventions to improve healthcare, their impact on practice and policy is limited so far [25]. Establishing and conveying the credibility of computational modelling and simulation outcomes is a delicate task [26] and the step from simulation to implementation in practice turns out to be a difficult one.

Kessler & Glasgow point out that healthcare research must deal with “wicked” problems that are multilevel, multiply determined, complex and interacting. Research tends to isolate, decontextualize and simplify issues in order to be able to investigate them. Consequently, the small number of studies with representative populations, staff and settings that substantiate optimisation approaches is in sheer contrast with the large number of papers that promote the potential of computational methods.

Overall, similar to what Fong et al. (2016) report, timepoints, cost, methodology and outcome measures were inconsistent across the studies in this review, and it appears that multiple metrics can fit a topic. Nevertheless, the topics of the articles cited in this review give insightful handles of how to structure OR performance metrics. Increasing awareness about these topics and metrics amongst the people who work with them is therefore of value.

Awareness should also be increased about the definitions of the concepts of OR performance [17]. It is important to realize that the term “*OR performance”* only describes a snapshot in time but extents across all topics. Some studies talk about performance, but do not always specify if there is change in this performance. Change can only be measured over time. When doing so, clear criteria are required to determine if the change is also an improvement. In one context something might be an improvement, in another it might worsen the situation [27].

The ideal scenario would be to optimize an OR performance topic for all the metrics from Table 3. However, this may not always be attainable. A sensible approach is to apply relevant metrics both at the beginning of your project and after your intervention in the system, and to evaluate the impact on all four categories of OR performance. By prioritizing and assigning weights to metrics acceptable ranges for the optimisation outcomes could be defined. When looking at optimization in this way, it should comprise two elements: improvement on a (set of) metric(s) and an improvement of the total system after your intervention.

**Fig. 4:**
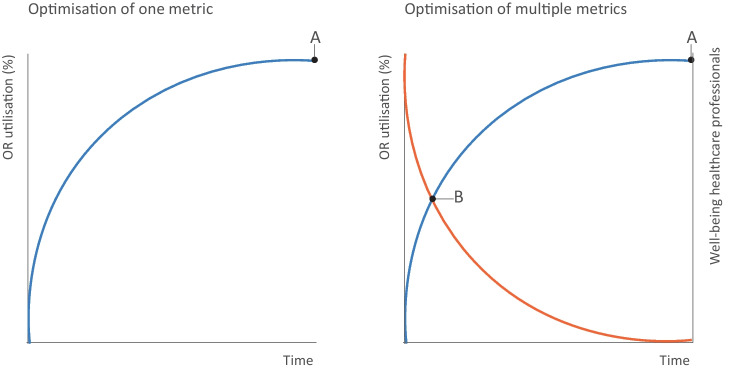
Two elements of OR performance optimization: optimize for a (set of) metric(s) and improve or conserve the overall balance of the metrics

This approach is illustrated in Fig. 4, where on the left the hypothetical optimization of one metric is shown, and on the right the same change of the metric is shown, together with another metric of the system. When, for example, one chooses to optimize OR performance by increasing the metric *Number of operations per OR per month*, you aim for point A in Fig. 4. However, Fig. 4 also illustrates that an increase along one metric could mean a (unintended) decrease on another. When taking other metrics into account you can see it is actually point B you are aiming for. Therefore, measuring every metric before and after your intervention to monitor the impact on the total system is essential for a thorough validation of its appropriateness.

### Modelling the metrics

**Fig. 5:**
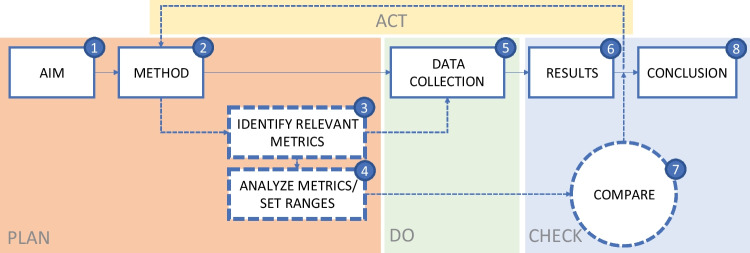
Suggestion for a research setup in which the whole system is taken into account by incorporating an analysis of the metrics. Based on the PDCA Cycle of Deming

Optimizing the system for a certain metric while also considering the other metrics should be part of the optimization strategy. Practical execution of this strategy is a roadmap with design steps in which the metrics are incorporated. In the following paragraphs this idea will be illustrated with Fig. 5 and an example scenario for an optimization goal.

Figure 5 shows an example of the main steps of a research approach (aim, method, data collection, results, conclusion) with an emphasis on the phase between method and data collection. The approach is based on the Plan-Do-Check-Act method of Deming [28]. In a fictitious scenario the aim of the project is to improve the well-being of healthcare professionals on the OR. This is the first step of the model in Fig. 5. In second step it is determined that the method used to achieve the aim will be increasing the metric autonomy of the healthcare professionals. A questionnaire amongst the staff involved shows that the healthcare professionals would like to have more autonomy over *when* they work. A more open work schedule is therefore suggested.

In the third step of the model the most important metrics that could be affected by this change are listed by the researchers:

- Excess staffing costs (often caused by over- or under-utilization of the OR).
- OR utilization
- Quality of care and patient safety
- Autonomy

In the fourth step the selected metrics are combined in logical sets as system optimisation metrics with assigned weights and acceptance ranges. As an example, we could look at optimizing OR utilization. In this case the constant consists of the metric *off time per OR per month* and the *utility time per OR per month* (see Eq. 1).

1 $$\frac{\displaystyle\frac{Toff}{\displaystyle\frac{OR}{month}}}{\displaystyle\frac{Tutility}{\displaystyle\frac{OR}{month}}}=\frac{Toff}{Tutility}=T\ast$$

Ranges of the constant are then given scores and weights to calculate the optimal value (Table 5). For example, if T* were to be greater than 1, there would be more off time per OR than utility time. That is an undesirable scenario. This range is therefore given a score of -1 and a weight of 2.

**Table 5:** The constant T* describes OR utility. To create insight in what values of T* are desirable and which are not, scores and weights are assigned to ranges of values of T*

	Score	Weight	
0 < T* < 0.25	2	2	Best
0.25 < T* < 1	1	1	Acceptable
1 < T*	-1	2	Worst

When T* is low there is a high utility rate of the OR’s. When T* is high there is a low utility rate. A more complete overview would be created when also plotting financial metrics and metrics concerning the well-being of the medical staff.

After data has been collected in step 5 of the model, the results of step 6 can be compared in step 7. One can then evaluate whether the intended innovation will improve the system in such a way that it is worth the investment. And if not, consider carefully based on the metric overviews where adjustments to the intervention are required.

### Limitations

This study has limitations. A major focus of this paper is the importance of seeing the whole picture when doing research. We have given examples of possibilities to bring this way of doing research into practice. However, despite the broad view of this study, we did not cover all aspects of healthcare. We looked at just the OR. Following our own philosophy, we want to stress that an even broader scope is relevant for successful optimization in healthcare. There is an intricate interplay between the different departments of a hospital. Increasing the efficiency of the OR might, for example, cause trouble in the timetable of the PACU (Post Anaesthesia Care Unit).

## Concluding remarks

In this study it was found that there are many different perspectives and approaches used to optimize OR performance. The metrics used to optimize OR performance are diverse. Based on our inventory of the metrics and methods used in literature we conclude that part of the crucial aspects of OR performance, such as the wellbeing of healthcare professionals, are underrepresented in the research field. The lack of studies that account for possible interactions between metrics of quality and efficiency have limited the impact of optimisation approaches. Too much focus on one metric potentially deteriorates other elements of the system you try to optimize. To obtain profitable OR optimization, a systems approach that aligns metrics across functions and better representation of the wellbeing of healthcare professionals are needed.

### Future research

An informative topic to investigate further is to test the effect of awareness of metrics when optimizing OR metrics in practice. The hypothesis here is that more awareness of OR performance metrics and their correlations amongst researchers could lead to better optimizing strategies. In this context, the model in Fig. 5 might also be tested. Does it increase awareness? Do researchers use different approaches with the model than without? Does this lead to better outcomes?

Another direction is the continuous measuring of OR performance metrics to be able to monitor unintended interactions, in ways that not put a burden on healthcare professionals (i.e., increasing administrative tasks). Furthermore, technology can speed up and smoothen processes within the OR, but the impact on perioperative processes might not have been considered. An interesting way to put these thoughts to practice is investigating how the increase of technology on the OR has influenced the work of healthcare professionals such as OR nurses and supporting department.

### Supplementary Information

Below is the link to the electronic supplementary material.

Supplementary file1 (PDF 106 KB)

Supplementary file2 (PDF 118 KB)

Supplementary file3 (DOCX 92 KB)

## Abbreviations

OR: Operating Room
FMEA: Failure mode and effects analysis
VSM: Value Stream Mapping
ER: Emergency Room
OSH: Occupational Safety and Health
PACU: Post Anaesthesia Care Unit
