# Supplementary figures and images for: Operating Room Performance Optimization Metrics: a Systematic Review
Source: J Med Syst. 2023 Feb 4;47(1):19. doi: 10.1007/s10916-023-01912-9 (PMC9899172; doi:10.1007/s10916-023-01912-9)

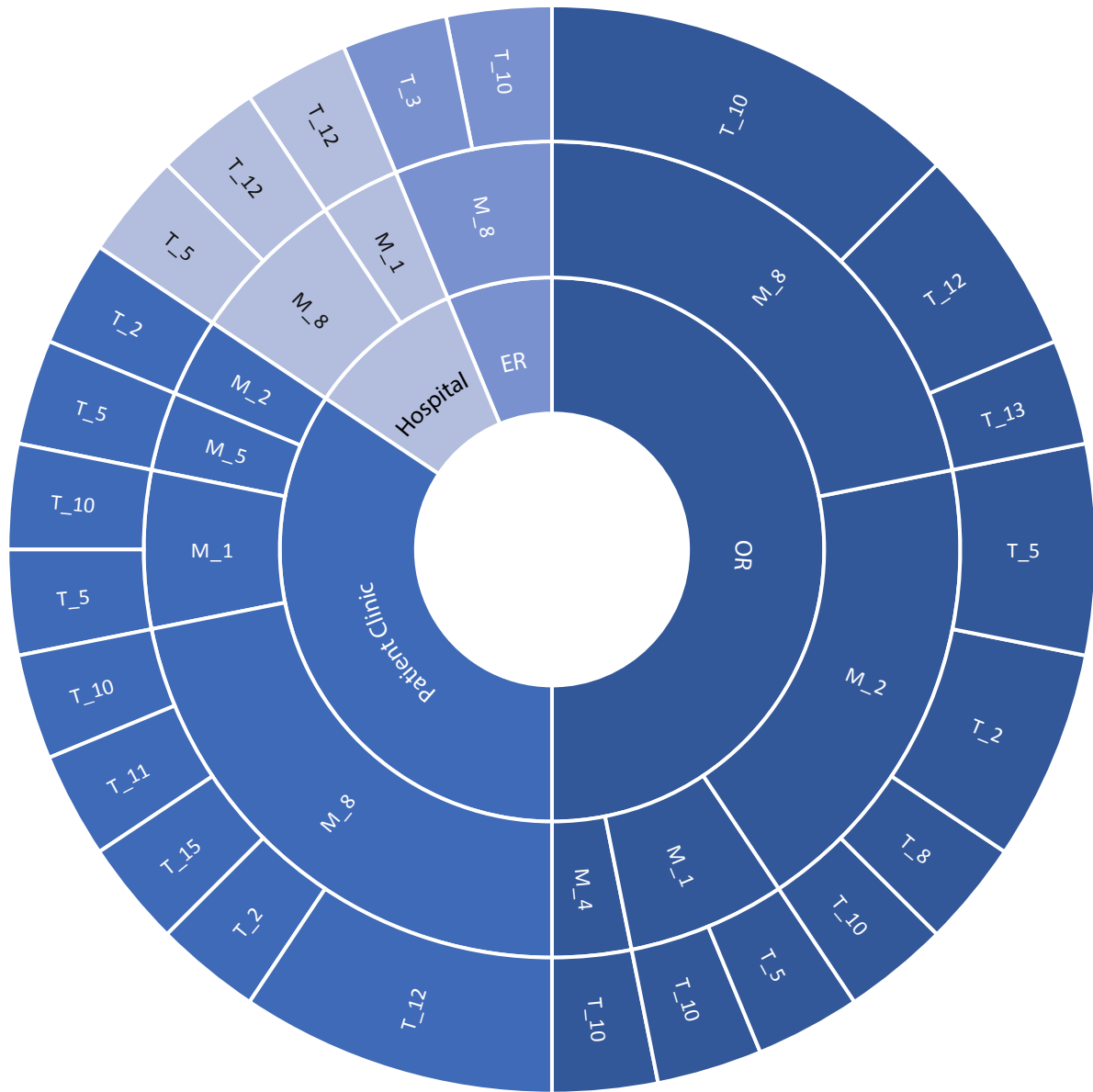

Supplement: Supplementary file 1 — Supplementary file1 (PDF 106 KB) [file 10916_2023_1912_MOESM1_ESM.pdf]

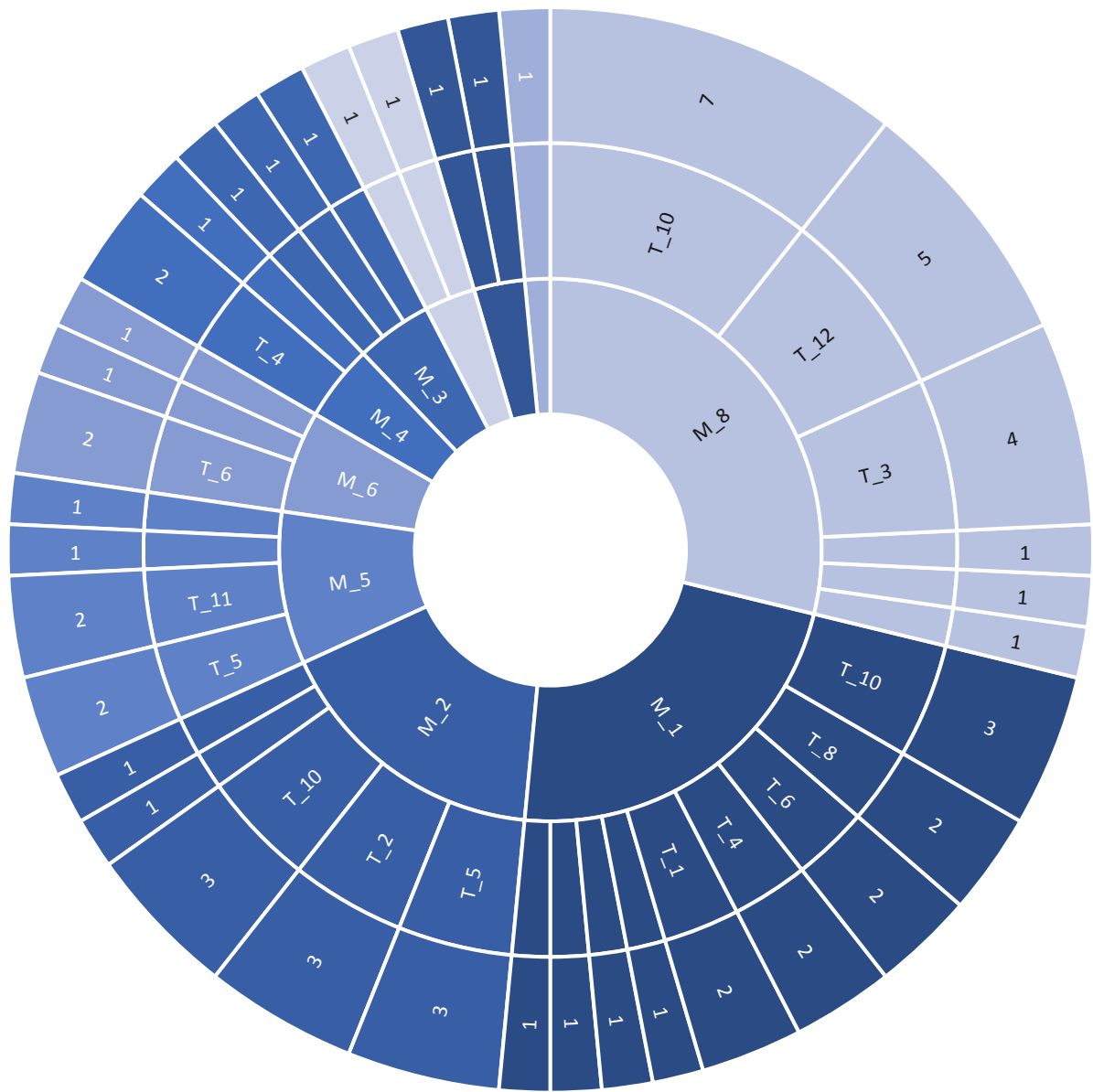

Supplement: Supplementary file 2 — Supplementary file2 (PDF 118 KB) [file 10916_2023_1912_MOESM2_ESM.pdf]
