# Supplementary material for: Operating Room Performance Optimization Metrics: a Systematic Review
Source: J Med Syst. 2023 Feb 4;47(1):19. doi: 10.1007/s10916-023-01912-9 (PMC9899172; doi:10.1007/s10916-023-01912-9)
Supplement: Supplementary file 3 — Supplementary file3 (DOCX 92 KB) [file 10916_2023_1912_MOESM3_ESM.docx]

Table 5 The identified metrics and their corresponding sources.

| *Label* | *Name* | *Source* |
| --- | --- | --- |
| T_1 | Optimize role of surgeon | (14) |
| T_2 | Reduce delays | (26)(27)(28)(29)(30)(31)(32) |
| T_3 | Optimize patient flow | (17)(5)(19)(33)(34)(35)(36)(37)(38)(39)(40)(41)(41)(42)(43)(3)(44)(45)(46) |
| T_4 | Reduce costs | (47)(8)(48)(49)(50)(51)(52) |
| T_5 | Optimize management | (53)(54)(55)(56)(57)(10)(58)(59)(60)(20)(61)(62)(63)(64)(65) |
| T_6 | Optimize teamwork | (66)(67)(68)(69)(70)(71)(72) |
| T_7 | Reduce non operative time | (73) |
| T_8 | Optimize anaesthesia procedure | (74)(75)(76)(77)(78)(79)(80) |
| T_9 | Define OR efficiency | (16) |
| T_10 | Optimize scheduling | (18)(81)(82)(83)(84)(85)(86)(87)(88)(89)(90)(91)(92)(93)(94)(95)(96) |
| T_11 | Optimize overall equipment effectiveness | (97)(98)(99)(100)(101) |
| T_12 | Optimize workflow tracking systems | (102)(103)(104)(105)(106)(107)(108)(109)(110)(111)(112) |
| T_13 | Optimize overall productive capacity of a department | (113)(114) |
| T_14 | Optimize department design | (115)(116)(117)(118)(119) |
| T_15 | Reduce workload | (120)(121) |
